# Filling Molars That Require Devitalization

**Published:** 1906-06-15

**Authors:** P. F. Hines


### FILLING MOLARS THAT REQUIRE DEVITALIZATION.

BY P. F. HINES, D.D.S.

Read before the Central Michigan Dental Society, February 22, 1906.

In presenting this paper to you to-day, it is not done with the view of giving anything especially new or original, but rather with the idea of giving you my method of using and experience with that which has been worked out and tried by others long before me. We all at times have patients presenting themselves to us, saying that they have the tooth-ache and upon a careful examination we find a molar containing a living pulp, and that caries has so far invaded the tooth as to cause pulpitis, and in the judgment of the operator it will be necessary to devitalize the pulp in order to restore the tooth to permanent quiet and future usefulness.

In such a case, or if for any other reason it becomes really necessary to devitalize the pulp of a molar, I would proceed as follows: First isolate the tooth by means of the rubber dam, after which dry the cavity with a pledget of cotton, carefully chiseling away overhanging frail margins, and remove with sharp, suitably shaped spoon excavators as much debris and soft decay as is possible, without inflicting too much pain upon the patient. Now dip a small pledget of cotton into a saturated aqueous solution of cocain containing ahout eight grains of boracic acid to the ounce, and place this pledget of cotton upon the exposed pulp, then cover the same with a ball of unvulcanized rubber sufficiently large to approximately fill the cavity, and then, with a round-headed burnisher, or other suitable instrument, bring to bear upon this gentle and gradually increasing pressure in such a manner if possible as to confine the liquid within the cavity.

In from one to three minutes you should be able to kneed the rubber into the cavity with firm pressure without inflicting the slightest pain. If not, remove from the cavity the rubber and cotton, when you can generally painlessly remove nearly all the soft leathery decay overlying the pulp, after which repeat the above operation, quickly securing complete anesthesia of the pulp. By means of sharp burs, excavators and the chip blower, remove all decay from the cavity and wipe out same with a pledget of cotton saturated with a strong alcoholic solution of carbolic acid. Then open into the pulp chamber and remove the bulbus part of the pulp, leaving intact that portion which is in the root canals.

If there is hemorrhage, this can usually be checked by a pledget of cotton dipped in cold water and placed in the pulp chamber a moment, after which remove and follow by another saturated with peroxide of dydrogen. If this fails as it does occasionally in cases of excessive secondary hemorrhage, spread out a small amount of absorbent cotton and place upon it as much dry tannic acid as can be rolled up and held by the cotton. Pack this into the pulp chamber and let it remain for a few moments and this will effectually check the hemorrhage. Wipe the cavity and pulp chamber with a pellet of cotton saturated with carbolic acid, dry and fill the pulp chamber nearly full of paste made after the following formula:

R Alum, dessicated.
Thymol.
Glycerol, aa, ʒ j.
Zinc oxide, q. s., to make a stiff paste, M.

This formula is the one successfully used by Dr. Soderberg and printed in the *Dental Cosmos*, May, 1896.

Seal the pulp chamber with cement and fill the cavity with amalgam, gold or any filling material most suitable for the place.

In attempting to follow this mode of treatment, be sure that the tooth in question contains a living pulp and not a gangrenous one, else your efforts will likely prove a great annoyance to your patient and a source of chagrin and embarassment to yourself. You will note in the method outlined that the same care and aseptic precautions are necessary as for root fillings or any other operation involving the pulp tissue.

In a period covering a little over five years, I have so treated 361 teeth and have yet to see one case of abscess or any failure to report. A large number of these patients visit my office from time to time and I have seen a great many of these teeth, and know that they are quiet and doing good service.

I have not extended this mode of treatment to the ten anterior teeth because of the ease with which the pulp can be removed and the canals successfully filled; and if later in life it becomes necessary to place upon them some form of post crown, the root is in readiness to receive the crown.

The chief advantage in this method over the orthodox method of removing the pulp in its entirety and filling the root canals are the certainty of good results, the operation can be completed at one sitting, less time is consumed, hence less fatigue to the patient, and I might say, to the dentist as well. The patient's time may be valuable, ours should be. The saving of tooth structure which would necessarily have to be cut away in order to gain access to the root canals, is a decided advantage as in the case of buccal cavities where the corronal portion of the tooth would necessarily have to be cut away, making possible the saving of second and third molars having crooked roots and cavities which are difficult of access. Discoloration of tooth structure is avoided, as the tooth retains its life-like appearance. For nervous patients valuable teeth for health and future usefulness, which might otherwise be lost, are easily saved, and it is done so nicely that when it becomes necessary for the patients to return to our office for other work, they do so without fear and trembling, greeting us with a smile instead of an expression of terror.

